# Event-related potential evidence that working memory whether inside or outside a virtual reality environment can reduce the extent of attention capture by irrelevant novel stimuli

**DOI:** 10.3389/fnins.2026.1654765

**Published:** 2026-02-18

**Authors:** Farooq Kamal, Nusrat Choudhury, Alexandra Doiron, Duncan Sadorsky, Kenneth Campbell, Cassandra Morrison

**Affiliations:** 1 National Research Council Canada, Boucherville, QC, Canada; 2 Department of Psychology, Carleton University, Ottawa, ON, Canada; 3 School of Psychology, and Brain and Mind Institute, University of Ottawa, Ottawa, ON, Canada

**Keywords:** auditory deviants, DRN, event-related potentials, MMN, P3a, switching of attention, virtual reality, working memory

## Abstract

**Introduction:**

If an individual is engaged in a visual task, the onset of a highly novel but unattended auditory stimulus may result in a switch of attention away from the processing of the task-at-hand and to the processing of the more potentially relevant auditory stimuli. This switch is called attention capture. An auditory deviant, representing a change to any feature of a frequently occurring standard stimulus, will automatically elicit an event-related potential, the deviant-related negativity (DRN). If the deviant is highly novel, it may also elicit a later positivity, the P3a, associated with the switching of attention. There is some evidence that carrying out a visual working memory (WM) task may reduce the extent of attention capture. Also, individuals carrying out a task within a virtual reality (VR) environment often report that they may not be aware of irrelevant external stimuli occurring in the external environment that might otherwise elicit attention capture.

**Methods:**

Nineteen young adults were engaged in three visual tasks: watching a silent video (control), performing a delayed match-to-sample WM task in a VR environment and performing a somewhat similar WM task presented on a 2D monitor. A multi-feature auditory sequence was presented concurrently but this was irrelevant to the visual task and was to-be-ignored. The sequence consisted of a frequently occurring standard stimulus and six different rarely occurring deviants, created by changing a different feature of the standard.

**Results:**

All unattended auditory deviants elicited a significant DRN, reflecting robust automatic detection of auditory change. The nature of the visual task had no significant effect on the DRN. Only highly novel deviants (white noise, environmental sounds) elicited a P3a when participants watched the video. This P3a was significantly reduced during both the VR WM and 2D WM tasks.

**Discussion:**

These findings suggest that early processes associated with detection of acoustic change operate automatically, regardless of the demands of the visual task. On the other hand, the P3a, associated with attention-capture and the switching of attention from the task-at-hand, was reduced in the VR a WM task. It was, however, also reduced in the 2D WM task. It is thus not clear whether the VR environment or the fact that participants were engaged in a WM task was responsible for the reduction of the P3a.

## 1 Introduction

A primary role of a frontoparietal central executive network is to establish processing priorities and to focus attention on those cognitive tasks deemed to have high priority. Noisy environments may interfere with and disrupt the ability to focus attention on the task-at-hand. There is good evidence that brain networks can adapt or habituate to continuous background noise (Sussman and Winkler, 2001). On the other hand, it is much more difficult to inhibit the impact of infrequently occurring, brief-lasting transient acoustic stimuli. Even though a task may be deemed to have high priority for processing, certain auditory transients occurring within the environment but outside the current focus of attention will nevertheless be involuntarily processed, causing an interruption of the central executive. This interruption, known as attention capture, or involuntary attention, results in a switch of attention from the processing demands of a current cognitive task and toward the processing of a potentially much more relevant auditory event (James, 1890). Most unattended input is however incidental. Thus, the switching of attention to these inputs does come at a cost. During attention capture, processing resources are switched to environmental input that is irrelevant to the task-at-hand; therefore, performance on the relevant cognitive task may deteriorate. This process is called distraction and is an obstacle to task execution (Lavie, 2005; Forster and Lavie, 2008; Parmentier, 2014). The present study examines how the extent of attention capture can be reduced.

Attention capture is often associated with auditory rather than visual stimuli (Morrison et al., 2020; Parmentier, 2014; Schröger, 1996). The use of auditory stimuli is because we hear auditory stimuli over a 360° space (in front of, beside, and behind our head) whereas vision is restricted to seeing stimuli in front of our head. Several behavioral studies have attempted to determine the extent to which unattended and irrelevant auditory distractors are processed (Escera et al., 2000; Parmentier et al., 2008). Behavioral performance on an assigned visual task might thus be poorer when unattended and irrelevant stimuli are presented compared to when they are not presented (Parmentier, 2016; Berti and Schröger, 2003; SanMiguel et al., 2010). Note that in these behavioral studies, the extent to which the irrelevant stimulus has been processed can only be implied based on performance. Directly quantifying the extent of processing of a to-be-ignored stimulus is difficult because the participant is not actively responding to it. The study of automaticity in information processing is facilitated by the recording of event-related potentials (ERPs), as they provide a means of determining the extent that to-be-ignored stimuli are processed. ERPs are the minute changes in the electrical activity of the brain that are elicited by a physical stimulus or an internal, psychological event. The ERPs consist of a series of negative- and positive-going components thought to reflect different aspects of information processing.

Auditory attention capture is often studied using a so-called oddball paradigm, consisting of a series of a frequently occurring “standard” stimulus and at rare (or “odd”) times, a “deviant” is created by changing a feature of the standard. The participant is often asked to focus attention on a visual task and to ignore the auditory channel. The auditory stimuli are therefore irrelevant to the visual task. Priority of processing is thus given to the visual task. As such, processing of the unattended auditory stimuli can only occur passively. Both the standard and deviant auditory stimuli will elicit an obligatory negative ERP component, N1, maximum in amplitude over frontocentral areas of the scalp. N1 occurs at about 100 ms after stimulus onset followed by a later positivity, P2 occurring at about 180–200 ms. The deviant elicits an additional frontocentral maximum negativity, the mismatch negativity (MMN) occurring between 100 and 200 ms. The MMN is elicited by any change in a feature of the standard stimulus, including its frequency, intensity, duration or location (Näätänen, 1990). In the original Näätänen model, a change detection system stores the extracted features of all auditory input in a rapidly fading sensory memory. With each occurrence of the standard, its representation in sensory memory improves. When a deviant is presented, at least one of its features fails to match that of the stored representation of the standard, and change is detected. The output of the change detection system is reflected in the MMN. A more recent model claims that the MMN does not necessarily reflect detection of a physical change to the standard stimulus but rather a violation of predictions formed based on the pattern or rules of stimulus presentation (Näätänen et al., 2011; Paavilainen, 2013; Winkler, 2007; Winkler et al., 2009). The oddball sequence is thus a special case in which the occurrence of a deviant violates the expectation for the presentation of the homogeneous, frequently-occurring standard stimulus. The MMN is thought to reflect a pre-attentive, pre-conscious detection of acoustic change. Thus, acoustic change is detected prior to awareness that a change of the auditory stimulus has occurred. Several studies have now indicated that the MMN can be robustly recorded following the presentation of a deviant stimulus regardless of the direction or strength of attention (Muller-Gass et al., 2005, 2006; Sussman, 2007, 2017; Hsu et al., 2023) or task demands (Ritter et al., 1999).

The MMN is best-observed in a deviant-standard difference wave. When the deviant is created by making only a small change to the standard (e.g., a 1,000 Hz standard and a 1,050 Hz deviant), the onset of both stimuli should elicit a very similar N1. The subtraction process will remove processes that are common to both the standard and the deviant (e.g., the N1), leaving only processing that is unique to the deviant, the MMN. The standard and deviant do not always elicit identical N1s. A deviant that represents an increase in auditory intensity or is highly novel (a stimulus in which a number of features change) will elicit a larger N1 than the standard. This larger N1 will therefore not be completely removed in the subtraction process. Because the novel deviant stimulus also signals change from the standard, it will also elicit an MMN. Unfortunately, the N1 and the MMN may overlap both temporally and spatially (they occur at similar time and are both maximum in amplitude over fronto-central areas of the scalp), resulting in the summation of the two negativities. What is observed in the deviant-standard difference wave is thus a large negativity representing a composite N1 + MMN, rather than a “pure” MMN. This composite negativity has been labeled as a deviant-related negativity or DRN (Alho et al., 1992; Muller-Gass and Schröger, 2007; Macdonald and Campbell, 2013; Tavakoli and Campbell, 2016). This convention will be used in the present study especially when a deviant is created by possible intensity change.

The rarely occurring deviant stimulus might also elicit a later centro-frontal maximum positivity, the P3a, peaking from 200 to 300 ms. While almost any perceptible change will elicit a DRN/MMN, only highly novel deviants will elicit the P3a when the auditory stimuli are to-be-ignored and are irrelevant to the task-at-hand (Polich, 2007). These deviants include environmental sounds and white noise whose frequency spectrum and stimulus energy (e.g., intensity) vary widely from the standard stimulus. It is the P3a that is thought to reflect processes associated with the involuntary switching of attention from the task-at-hand and toward the processing of the unattended acoustic change (Escera et al., 1998; Parmentier, 2014; Wetzel et al., 2013). The switching of attention to the auditory channel and the need for additional processing of the highly novel, salient auditory stimulus may then lead to eventual consciousness of it. Whether the P3a reflects the actual switching of attention (Escera et al., 1998) or processes that may lead to the switching of attention (Wetzel et al., 2013; Parmentier, 2014) remains disputed.

Several studies have examined how distraction by auditory stimuli resulting in deterioration in performance on a variety of tasks can be prevented by, for example, varying processing task demands (see reviews by Lavie, 2005, 2010). A perceptual task that is very difficult will make more demands on the limited processing resources, leaving few for the co-processing of other irrelevant stimulus input. On the other hand, an easy perceptual task will leave many resources available for the co-processing of these irrelevant inputs. In general, when a perceptual task is relatively easy, distractors will have a larger effect on performance than when the task is particularly difficult. The results are nevertheless dependent on several factors including the nature of the task (perceptual versus working memory) and within-modality versus cross-modality interference (see reviews by Lavie, 2005, 2010).

While there is general agreement that task demands have minimal effects on the MMN, there is some dispute about the extent to which the P3a is modulated by similar task demands. Initial studies of the auditory P3a indicated that it was largely an automatic process and its amplitude did not vary between easy and difficult visual tasks. For example, Muller-Gass et al. (2007) had participants engage in a continuous visual object tracking task which varied in difficulty. An auditory sequence was presented concurrently but it was irrelevant to the visual object tracking task. The continuous nature of the visual task was designed to prevent participants from sampling the auditory sequence, particularly when the task was very difficult. Thus, participants should not have been able to listen to (eavesdrop on) the auditory sequence while also attending the visual task. An auditory deviant created by increasing the intensity of the standard elicited a large P3a, but importantly this P3a was not affected by task demands. Muller-Gass and Schröger (2007) employed a two-stimulus duration detection task, participants being asked to determine the duration of the auditory stimuli. At rare times, a feature of the auditory stimuli was changed but this was irrelevant to the duration discrimination task. This deviant elicited a P3a, whose amplitude did not vary between easy and difficult duration detection conditions. Similarly, Volosin and Horváth (2020) also used an easy and difficult duration discrimination task and observed that task difficulty did not affect the amplitude of the P3a.

Some studies have employed working memory (WM) tasks to investigate whether cognitive load can protect against auditory distraction. It has been well-established that the maintenance of items in WM requires active attention (Baddeley and Hitch, 1974; Cowan, 1995). Thus, the high WM load uses the cognitive resources that would otherwise be available for the processing of irrelevant auditory input. Most studies have used an n-back task to assess WM. In the *n*-back task, the participant is asked to determine whether the current stimulus matches a stimulus presented *n* trials earlier in the sequence. In the Muller-Gass and Schröger (2007) study, a 1-back memory task condition was also run. Participants were asked whether the present short or long duration auditory stimulus was the same duration as the one that had preceded it. The pitch of the frequently occurring standard was at times changed to form a deviant, but the pitch change was irrelevant to the 1-back memory task. The distractor deviant resulted in poorer memory performance. These performance results were similar to those reported by Lavie (2005). In addition, a larger P3a was elicited when the participant had to decide whether the duration of the current auditory deviant was also presented in the previous trial (1-back condition) compared to when the participant had to decide about its duration (0-back condition). Thus, the *n*-back task seemed to *enhance* rather than protect against distraction. The effects of a distractor depend on several factors. In the Muller-Gass et al. (2007) study, the auditory distractor occurred within an auditory *n*-back task. Other studies have used a visual *n*-back. In these studies, auditory stimuli occur prior to visual stimuli, but they are irrelevant to the task (Lv et al., 2010; SanMiguel et al., 2008). These studies have reported that the P3a elicited by irrelevant deviants was reduced in amplitude when the *n*-back task was more demanding (Lv et al., 2010; SanMiguel et al., 2008). On the other hand, Mahajan et al. (2020) did not find that *n*-back task difficulty had a significant effect on the amplitude of the auditory P3a. In the *n*-back studies, the presentation of an irrelevant auditory stimulus prior to the relevant visual stimuli is problematic. While the auditory stimuli were irrelevant to the visual *n*-back, they could still have been used as a warning signal or as a cue to predict the subsequent occurrence of the visual target (Baragona et al., 2025; Parmentier, 2014). Thus, attending to the auditory sequence could have been used to improve performance on the visual WM task. As such, differences in the amplitude of the P3a may have been a result of passive compared to active processing of the deviant.

The manipulation of task demands is based on the assumption that the strong attentional focus required by a difficult task does not allow participants to also attend to and process the irrelevant auditory input. By contrast, during an easy task, additional attentional resources should be available to process the irrelevant auditory input. Muller-Gass et al. (2006) have however provided behavioral evidence that even during very difficult visual tasks, participants might still be able to actively attend to auditory input. They employed easy and difficult visual discrimination tasks while auditory stimuli were presented concurrently. In one condition, participants were required to overtly divide their attention between the auditory and visual channels and asked to detect rarely occurring targets in both modalities. Even when the visual task was quite difficult, when participants were asked to divide their attention between the two channels, they were able to successfully detect the rarely occurring visual *and* auditory targets. Thus, even when an auditory sequence is to-be-ignored, it may nevertheless be possible to eavesdrop on the auditory channel and still maintain a high level of performance on the visual task, even if the visual task is difficult and makes high demands on attentional resources.

Recent technological advances such as virtual reality (VR) offer a newer method to manipulate task engagement. The realistic 3D VR environment has been described as being highly “immersive” and thus extremely demanding of cognitive resources (Al Boustani et al., 2022). Individuals often experience an intense sense of “presence” such that the virtual environment becomes the dominant reality. These individuals may even be unaware of their real, external world (Sanchez-Vives and Slater, 2005). Engaging in a VR task may therefore be able to prevent the switching of attention to the irrelevant auditory input occurring in the external environment. The present study examines whether the amplitude of a P3a elicited by the occurrence of an unattended rare, novel deviant stimulus can be reduced by carrying out a visual WM task within a VR environment. Perhaps the most convincing evidence that VR tasks can be extremely demanding of cognitive resources comes from studies of how an extremely salient, but task-irrelevant stimulus, pain, can be inhibited (see Hadjiat and Marchand, 2022 for a review). Engagement in a visual VR task has been shown to be associated with a reduction in participants’ rating of both acute and chronic pain (Viderman et al., 2023; Teh et al., 2024; Nagamine, 2025). Such ratings are however very subjective and prone to bias. ERP responses to unattended painful stimuli have been used to provide an objective measure of the extent to which these stimuli are processed. Lier et al. (2020) asked participants to watch video scenes within a VR environment. Painful electric shocks were presented occasionally but these were irrelevant to the VR task. In a control condition, participants saw a static image and again, the irrelevant electric shocks were presented. The pain stimulus elicited a late positivity occurring between 220 and 250 ms. This positivity is consistent with the P3a. The P3a following presentation of the electric shock was reduced in amplitude when the participants were actively engaged in watching the video compared to when they saw the static image within the VR environment.

In other studies, irrelevant auditory rather than pain stimuli have been presented. The results however vary; some studies show an effect of engaging in a VR task such that the amplitude of the ERPs to the irrelevant auditory stimuli are reduced, while others do not show the effect (Al Boustani et al., 2022; Burns and Fairclough, 2015; Chen et al., 2014; Grassini et al., 2021; Kober and Neuper, 2012; Sarasso et al., 2024; Terkildsen and Makransky, 2019). VR task demands and auditory stimulus parameters vary widely across studies, making generalization difficult. Moreover, in the oddball studies that have been run, the auditory deviant stimulus may not have been sufficiently salient to elicit a P3a, and induce a switch of attention from the VR task.

The present study employs a WM task adapted to the VR environment. A VR version of the *n*-back task using its highly realistic 3D capabilities has yet to be implemented. Climent et al. (2021) and Klotzsche et al. (2023) have developed a VR version of a delayed match-to-sample task. In this type of WM task, on a single trial, participants may see a different number of items and after a delay are presented with a probe. Participants are asked whether the probe matches what has previously been presented. These tasks were, however, designed to closely mimic procedures used in traditional 2D WM tasks. Kamal et al. (2025) have described the development of a dynamic realistic 3D version of a delayed match-to-sample task. They demonstrated that it had essential attributes common to 2D versions of a WM task. Increasing the number of items to be remembered (i.e., WM load) resulted in poorer performance. WM capacity was also poorer in older than younger adults. It is this task that will be used in the present study.

An issue with the use of VR tasks is that some individuals are often unable to tolerate lengthy testing periods. Many auditory studies employ an oddball paradigm that usually only includes one or two rarely occurring deviants within the sequence. Most of these studies presented only a single deviant stimulus that was not sufficiently novel to elicit a P3a. To determine the effects of different deviants on the P3a, the usual oddball paradigm would need to be repeated several times, one time for each deviant, leading to a lengthy testing period. This long testing time may not be possible within VR environments. However, may be overcome using a time-efficient, multi-feature paradigm (Näätänen et al., 2004). This paradigm allows for the presentation of several deviants in a single run, each representing a change of a different feature of the standard. The sequence consists of an alternating pattern of standards and deviants. While the overall probability of standard and deviant occurrence is 0.5 for each, the probability of any specific type of deviant is lower. If five deviants are presented, each different deviant occurs relatively rarely, on 10% of trials. Tavakoli and Campbell (2016) developed a multi-feature paradigm designed specifically for the study of the P3a, in which six different deviants were presented in a single sequence. All deviants elicited a DRN. Only a white noise and novel environmental sound deviants elicited a P3a, while other deviants representing frequency, intensity (both increases and decreases), and duration changes did not.

A multi-feature paradigm was therefore used to efficiently record ERPs to many different types of auditory deviants. In most DRN/MMN studies, participants are asked to watch a sub-titled silent video while ignoring the auditory sequence containing the standards and deviants. The use of this visual task is based on the rationale that the DRN/MMN is largely unaffected by task demands and what the participant “is doing.” However, Muller-Gass et al. (2006) have provided strong evidence that participants can, in fact, also co-monitor the auditory channel while carrying out this visual task. Two other visual tasks were run. In a second condition, participants were engaged in a delayed match-to-sample WM task within a VR environment. The VR environment may be so demanding of cognitive resources that few resources will be available to allow for the co-monitoring and processing of the auditory channel. A third condition was also run. Participants were also engaged in a somewhat similar delayed match-to-sample WM task outside of the VR environment. This task was presented on a 2D computer monitor (thus a 2D WM task). If the VR environment is highly demanding of cognitive resources, then attention capture and the P3a should be reduced compared to the control condition. It is however possible that simply engaging in a WM task itself will require many processing resources. As such, the P3a may be reduced in both the VR and 2D versions of a WM task.

## 2 Materials and methods

### 2.1 Participants

Twenty young adults volunteered to participate in this study. All reported normal hearing. One participant was excluded from analysis because of noisy EEG data (see section 2.4). A total of 19 young adults’ aged 18–30 years were therefore analyzed (7 males). All participants were right-handed, with no history of neurological or psychiatric conditions. None were taking medication that could influence central nervous system functioning. This study was approved by the National Research Council of Canada and Carleton University (clearance number 121501) Ethics’ boards following the guidelines of the Canadian Tri-Council on ethical conduct involving humans. Participants provided informed written consent before starting the study and an honorarium was provided.

### 2.2 Stimuli and procedure

Participants were asked to focus attention on three different visual tasks presented in different conditions. The order of conditions was randomized. Auditory stimuli were presented concurrently but were irrelevant to the visual tasks and were to-be-ignored. In all conditions, participants were seated in a non-swivel chair in order to reduce muscle artifact. Participants were asked to avoid blinks and overt eye and body movements as much as possible.

#### 2.2.1 Passive video control task

In condition one, participants watched a silent English sub-titled Planet Earth video. It was presented on an 8.8 inch (approximately 22 cm) tablet at a distance of about 1 m from the participant. Watching a video is a commonly used task in DRN/MMN and P3a research and therefore served as a control condition. This condition was repeated twice, with each block lasting about 10 min.

#### 2.2.2 Virtual reality working memory task

In the second condition, participants were engaged in a delayed match-to-sample WM task within a realistic 3D VR environment. During the VR WM task, participants wore a head-mounted Meta Quest 2 VR headset, having a 1,832 × 1,920 pixel Fast-Switch LCD display, a 90 Hz refresh rate, and an 89° field of view. The VR system employed wireless hand-held controllers, each featuring a grip handle and a tracking ring at the top for positional and motion tracking. The controllers were equipped with a primary trigger and a grip button, enabling participants to actively interact with and grasp virtual objects.

**FIGURE 1:**
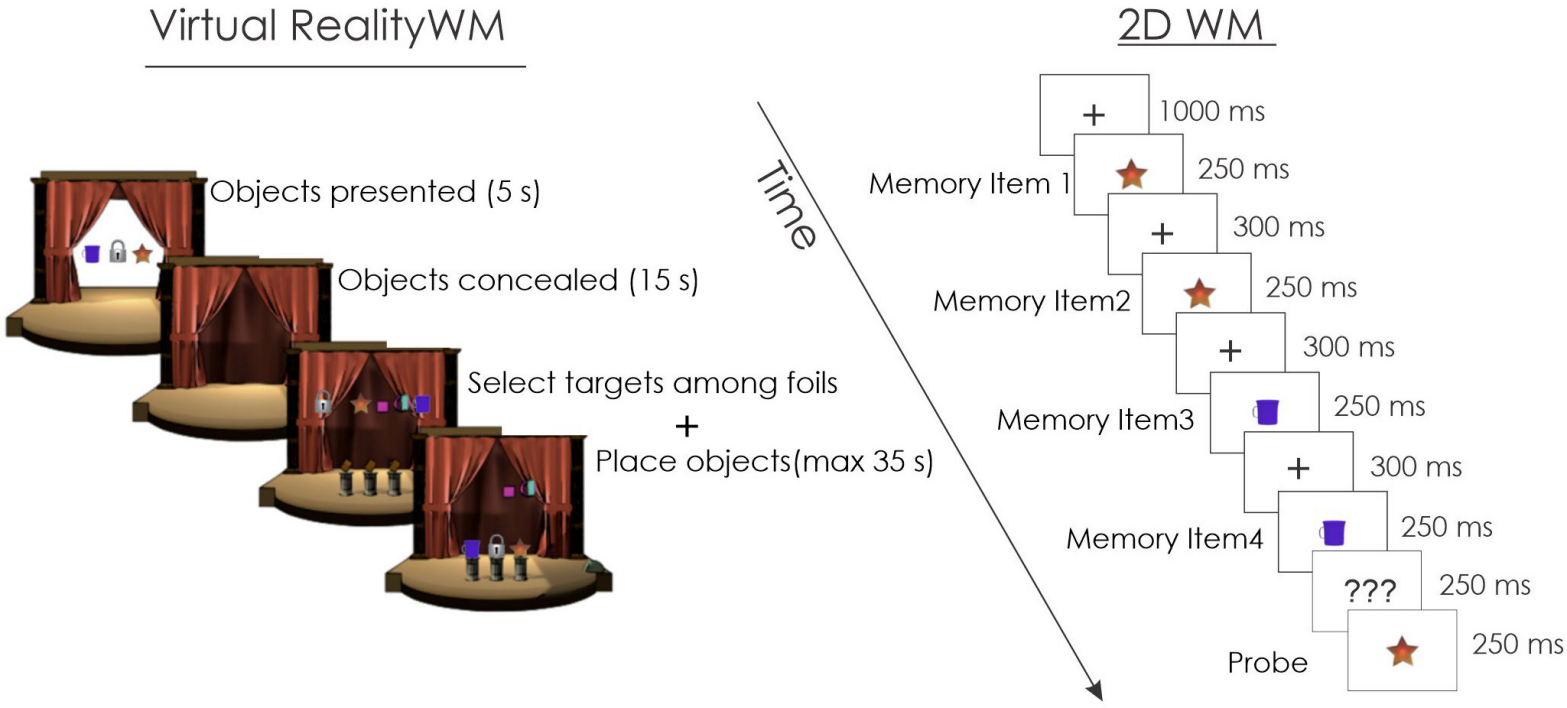
Delayed match-to-sample working memory (WM) tasks. In the left portion, the procedures for the virtual reality (VR) WM task are illustrated. In this example, the participant was presented with 3 target objects in the encoding phase. In the subsequent recognition phase the same 3 targets and additional foils were presented. The participant needed to select the 3 targets that were originally presented and place them on a pedestal. In the right portion, the procedures for the 2D WM task are illustrated. The participant could be asked to remember 1, 2, or 4 objects. The objects were presented sequentially. In this example, 2 objects were presented and repeated twice within the 4 object presentations. A probe was then presented and the participant needed to decide whether this probe was among the objects that had been previously presented.

The VR version of the WM task was adopted from bWell, a multisensorial platform developed by the National Research Council of Canada (Shaigetz et al., 2021; Kamal et al., 2025). The task is similar to a traditional delayed match-to-sample WM task and thus includes encoding, maintenance, recognition, and response phases. The procedure for the VR version of the WM task is illustrated in the left portion of Figure 1. Participants were placed in a realistic 3D theatre scene. In the encoding phase, participants saw different objects that appeared on the theatre’s screen for 5 s. Participants were instructed to remember these objects. The objects were subsequently concealed behind a curtain for 15 s. During this maintenance phase of the task, participants needed to retain the encoded objects in memory. All objects could be converted to a verbal code (or “named”) (e.g., heart, triangle) permitting the use of articulatory rehearsal to maintain the encoded objects in memory. In the subsequent recognition phase, objects that matched and others that did not match the previously presented objects (i.e., foils) fell to the stage. Participants had to identify the matching objects and use the controller to pick them up and then place the objects on a pedestal in the correct sequence. The participants were then asked to push a green button that appeared on the stage thus completing the trial. The time to complete the recognition and response phase was very long. They lasted until the response was executed. A 35 s time limit was imposed. Elaborate and precise hand and arm movements were required to execute the response. The next trial began 8 s after the execution of the response The complex and demanding response sequence further assured attention was focussed on the WM task, preventing sampling of the auditory sequence. The same number of objects appeared across five consecutive trials. Successful performance led to the addition of an object (step-up), while an error led to the removal of an object (step-down).

The VR WM task was repeated twice. Each block lasted about 10 min. Prior to the start of the initial VR WM block, participants were given a practice session lasting about 5 min to ensure they understood the instructions and response demands.

#### 2.2.3 2 D working memory task

In a third condition, participants were engaged in a similar delayed match-to-sample WM task presented on a 14 inch (approximately 35 cm) 2D computer monitor having a 90 Hz refresh rate, located about 1 m in front of the participant. The 2D WM task is illustrated in the right portion of Figure 1. In this task, participants were presented with a sequence of different objects followed by a probe. The objects that were used in the VR WM task were also used in the 2D WM task. The participant’s task was to determine whether the probe was a member of the set that had just been presented. Each trial began with a 1,000 ms duration fixation point (“ + ”) presented in the center of the monitor. The memory set was then presented. This memory set consisted of 1, 2, or 4 different objects. In each trial, 4 objects were always presented. When the memory load was 1, this object was repeated 4 times. When the memory load was 2, the first object was repeated 2 times followed by the second object, repeated 2 times. Four different objects were presented sequentially when the memory load was 4. The objects were taken from those used in the WM task within the VR environment and all objects could therefore be converted into a verbal code (i.e., were nameable). Stimulus duration was relatively brief, each image lasting 250 ms, followed by an interstimulus interval (offset-to-onset) of 300 ms. After the final image of the set, a question mark appeared for 250 ms. This question mark served to warn the participant that the probe was about to be presented. The duration of the probe was also 250 ms, but participants could respond up to 1,000 ms after presentation. Participants were asked to push one mouse button if they decided the probe had been a member of the set and a second mouse button if they decided it had not been previously presented. A total of 104 trials were presented. Because there were only a limited number of objects used in the VR, they were repeated at random over the 104 trials. Half of the probe objects were part of the memory set (positive probe), while the remaining half were not (negative probe). An equal number of positive probes were presented at random in either the first, second, third, or fourth position of the memory set. The memory set and probe objects subtended a horizontal visual angle of 15° and a vertical angle of 10°.

A total of 104 trials were presented, also lasting about 10 min. The 2D WM task was repeated twice. Each block also lasted about 10 min. Again, prior to the start of the initial block, participants were given a practice session lasting about 5 min.

### 2.3 Multi-feature auditory paradigm

A multi-feature auditory sequence was presented concurrently with each of the visual tasks. The auditory stimuli were created using Audacity, version 2.1.0 software. The auditory stimuli were however irrelevant to the visual tasks and were to-be-ignored. Auditory stimuli were synthesized using a SoundBlaster 16-bit waveform generator and presented binaurally through calibrated Sony MDR V6 over-the-ear headphones. These headphones have a relatively flat frequency response from 500 to 4,000 Hz. Standard stimuli (80 dB SPL, 1,000 Hz pure tone, 200 ms duration with a rise-and-fall time of 5 ms) alternated with 6 different deviants. The standards and deviants thus occurred on 50% of trials. The six deviants were created by changing a feature (or features) of the standard: a 10 dB intensity increase (increment), a 20 dB intensity decrease (decrement), a frequency change to 1,100 Hz, a duration decrease to 100 ms, a white noise burst, and environmental sounds. A different environmental sound was presented on each trial. The features of the environmental sounds are described in detail by Fabiani et al. (1996). A number of different sounds were presented including animal and human sounds, in addition to sounds commonly heard (telephone ringing, water dripping, car honks, etc.), musical instruments, and mechanical sounds. Although the standard:deviant probability of occurrence was 0.50:0.50, the probability of occurrence of a specific deviant was 0.083. In an array of 6 standards and alternating 6 deviants, each deviant occurred one time. The order of occurrence of a specific deviant was randomized. In each subsequent array, the order of occurrence of a specific deviant was again randomized. The same deviant was never presented consecutively (the first deviant in a new array could not be the same as the last deviant in the previous array). The first ten sounds in the sequence consisted of only standards to establish a memory trace for the standard stimulus. Stimuli were presented rapidly with a stimulus onset asynchrony of 600 ms. Each sequence lasted slightly more than 9 min with 472 standards (including 10 standards presented before the alternating sequence) and 77 of each of the 6 deviants being presented. The multi-feature sequence was presented twice for each visual task condition. A brief rest period was provided between blocks. Total testing time was about 90 min.

### 2.4 EEG recording

EEG activity was recorded using 29 active silver-silver chloride electrodes, attached to an electrode cap (Brain Products GmbH, Gilching, Germany) and placed over frontal, central, parietal, temporal, and occipital sites according to the international 10–10 system. Two additional electrodes were placed on the left and right mastoids (FT9, FT10), where the DRN/MMN inverts in polarity. An additional ocular (EOG) electrode was also placed on the infraorbital ridge of the left eye to record vertical eye movements and blinks. A reference electrode was placed on the tip of the nose. The EEG was sampled at a rate of 500 Hz (i.e., every 2 ms). The low-pass filter was set at 250 Hz. The time constant was 10 s (a high-pass filter of about 0.016 Hz). Electrode impedance was below 20 kΩ. Frontal (F3, Fz, F4) and central (C3, Cz, C4) sites were regions of interest (ROIs), where the DRN and P3a are maximum in amplitude. Impedances at these ROIs were below 10 kΩ.

The physiological data were subsequently analyzed using Brain Products’ Analyzer2 software. The EEG and EOG data were digitally filtered using a high pass filter of 0.5 Hz and using a low-pass filter set at 20 Hz (24 dB/octave roll-off). The EEG was visually inspected for channels containing high levels of noise. These channels were replaced by interpolating the data of the surrounding electrode sites (Perrin et al., 1989). Interpolation was not used for the frontal and central ROIs. Only 1 participant had more than 4 channels containing excessive noise and was thus excluded from further analyses. Independent Component Analysis (Makeig et al., 1996) was then used to identify eye movement and blink artifacts that were statistically independent of the EEG activity. To correct for these artifacts occurring within the EEG signals, vertical and horizontal EOG activity needed to be computed. A vertical EOG channel was computed by subtracting activity recorded at FP1 from the infra-orbital ridge. A horizontal EOG channel was computed by subtracting FT9 activity from that of FT10. The continuous EEG was then reconstructed into 700 ms epochs starting 100 ms before stimulus onset. The average of EEG within the 100 ms pre-stimulus period served as a zero-voltage baseline for all standard and deviant stimuli. Each single trial epoch was then baseline-corrected to remove slow voltage “drifts.” For each of the standard and deviant single trials, the mean amplitude of all data points within the baseline period was subtracted from that of all subsequent data points in the post-stimulus period. Any single epoch containing activity exceeding ± 100 μV was then rejected from further analysis. Rejections based on this criterion were relatively rare because the most common type of artifact, eye blinks and movements, had already been corrected. Other sources of artifact including arm and hand movements occurring within the VR task were largely attenuated by the EEG bandpass filter. Table 1 presents the total number of trials that were accepted for the different conditions and stimuli. Fewer than 1.5% of trials were rejected on the basis of artifact. Following the presentation of both standard and deviant stimuli, slightly more trials were rejected during the VR condition (1.3% for both standards and deviants) than during the 2D WM condition (0.5% of standards and 0.6% of deviants). While the differences between the conditions were statistically significant (*p* < 0.05 in both cases), such small differences had little practical significance. The single epochs were then sorted according to electrode site, visual task condition, and stimulus type (standard, six deviants), then averaged.

**TABLE 1:** Mean number and SD of accepted trials for the standard and deviant stimuli in the 3 visual task conditions.

Condition	Stimulus	Total trials	Accepted mean	Accepted SD
*Movie*	Standard	924	919.4	10.4
	Environmental	154	151.6	3.3
	White noise	154	152.6	2.3
	Decrement	154	152.7	2.0
*VR WM*	Increment	154	152.1	2.7
	Duration	154	152.1	1.9
	Frequency	154	152.3	2.2
	Standard	924	913.1	16.5
	Environmental	154	152.2	2.4
	White noise	154	152.2	2.2
	Decrement	154	152.2	2.1
*2D WM*	Increment	154	151.2	2.4
	Duration	154	151.9	2.8
	Frequency	154	152.2	2.2
	Standard	924	919.1	4.3
	Environmental	154	153.3	1.0
	White noise	154	153.2	0.9
	Decrement	154	153.2	0.9
	Increment	154	152.9	1.1
	Duration	154	153.3	0.9
	Frequency	154	152.9	1.1

#### 2.4.1 ERP Quantification and analysis

Both the standard and the deviant elicited obligatory N1 and P2 ERP deflections. The deviants elicited a series of additional deflections, including the DRN, and for some deviants, the P3a. The DRN and P3a are best observed in a difference wave computed by subtracting point-by-point the average response of the standard from that of the deviant. The older, traditional method for scoring ERP deflections identified a maximum peak within latency range. There are however both theoretical and methodological problems with this method. It assumes that a cognitive process, for example, change detection, occurs at a specific point in time. In reality, cognitive processes and decisions occur over a period of time. The peak detection method encounters issues when low amplitude responses are embedded in high frequency noise. Residual noise might thus be measured as the maximum peak. Luck (2014) recommends a different measurement technique. All data points within a specific interval of time are averaged. This procedure has the advantage that it better approximates the duration of an actual cognitive process. The mean amplitude measure should also cancel the positive- and negative-going residual noise. The DRN and P3a were therefore quantified for each individual using the mean amplitude of all data points within ± 25 ms of the peak identified in the grand average difference wave for each deviant and each task condition. There are problems with the mean amplitude measure. It may tend to smear or underestimate the true amplitude. Table 2 presents the 50 ms time intervals used for the scoring of the DRN and P3a in the control (watch movie) condition. Their amplitudes were measured with respect to the zero-voltage pre-stimulus baseline.

**TABLE 2:** 50-ms time windows used for the scoring of the DRN and P3a in the control (watch movie) condition.

Deviant	DRN	P3a
Environmental	095–145	185–235
White noise	085–135	180–230
Decrement	080–130	240–290
Increment	075–125	205–255
Duration	075–125	255–305
Frequency	075–125	210–260

The same approximate time windows were also used for the VR WM and 2D WM conditions.

Significant differences among deviant ERPs would usually be determined with an analysis of variance (ANOVA) statistical procedure. It was expected that only certain deviants would elicit a P3a but for other deviants, a P3a would be absent (Tavakoli and Campbell, 2016; Morrison et al., 2020). The usual ANOVA procedure could be used to determine if the P3a amplitude was significantly *smaller* or *larger* among deviants. The ANOVA cannot be used to determine if the P3a was absent (i.e., not elicited). To confirm if a DRN or P3a had been elicited by a deviant, confidence intervals were initially computed to determine if both the lower and upper limits were significantly less (or negative-going, in the case of the DRN) or greater (or positive-going, in the case of the P3a) than the zero-amplitude pre-stimulus zero voltage baseline. The procedure was run at the Fz electrode site for the DRN and at Cz for the P3a, where each tends to be at maximum amplitude. Because a directionality was predicted (negativity in the case of the DRN and positivity in the case of the P3a), one-tailed tests of significance (*p* < 0.05) were applied to the confidence intervals. Such use of a liberal statistical procedure does increase the risk of type I error, claiming a response was elicited when in fact, it was absent. The use of liberal procedures would allow possible small amplitude responses to pass to the additional ANOVA procedures. A more conservative procedure would remove the possibility of additional analyses. Nevertheless, to restrict the likelihood of chance findings, the negativity had to conform to the usual latency (100–250 ms) and scalp distribution (fronto-central maximum, inversion in polarity at the mastoids) for the DRN, while the positivity had to conform to the usual latency (200–300 ms) and scalp distribution (centro-frontal maximum) of the P3a.

Confidence interval testing for the DRN (Table 2) revealed that its amplitude was significantly different from zero-voltage baseline for all 6 deviants. Separate ANOVAs were then conducted at frontal (F3, Fz, F4) and central (C3, Cz, C4) ROIs and included the DRN data for all six deviants. A 3-way ANOVA with repeated measures on visual Task (video, VR WM, and 2D WM), Deviant (intensity increment, intensity decrement, frequency and duration change, white noise, and novel environmental sounds) and electrode site was computed for the DRN. Confidence interval testing for the P3a (Table 3) revealed that its amplitude differed significantly from the zero-voltage baseline for only the white noise and novel environmental sounds. The same 3-way repeated measures ANOVA was run but the deviant factor was restricted to the white noise and environmental sound deviants. The ANOVA was again run separately for the frontal and central ROIs. A 2-way ANOVA with repeated measures was also run for the N1 and P2 deflections following the presentation of the standard.

**TABLE 3:** Mean DRN, measured at Fz and P3a, measured at Cz, amplitude (SDs) and 95% lower and upper confidence intervals for the control (passive video watching) condition.

Deviant	Amplitude (SD)	95% CI
**DRN**		
Environmental	−1.65 (1.52)	(−2.39, −0.92)
White noise	−1.15 (1.55)	(−1.90, −0.41)
Decrement	−2.03 (1.32)	(−2.67, −1.40)
Increment	−1.42 (1.41)	(−2.10, −0.74)
Duration	−1.57 (1.03)	(−2.06, −1.07)
Frequency	−2.01 (1.13)	(−2.55, −1.46)
**P3a**		
Environmental	+6.11 (3.38)	(+4.49, +7.74)
White noise	+6.56 (2.85)	(+5.96, +8.71)
Decrement	+1.02 (1.31)	(−0.56, +1.84)
Increment	+1.32 (2.17)	(−0.43, +2.36)
Duration	−0.17 (1.63)	(−1.47, +1.35]
Frequency	−0.24 (1.57)	(−1.26, +1.15]

The assumption of sphericity for repeated measures was tested using the Mauchly procedure. When the assumption was violated (*p* < 0.05), Greenhouse and Geisser (1959) correction procedures were applied.

## 3 Results

### 3.1 Performance data

#### 3.1.1 2 D WM

Performance was measured in terms of accuracy of detection of the targets (hits) and foils (correct rejections), and speed of responding, reaction time (RT). A one-way ANOVA with repeated measures on Load (1, 2, or 4 items) was computed on these data. As expected, as WM Load increased from 1 to 4 items, the hit rate significantly decreased, *F*(2, 36) = 55.43, *p* < 0.001, η_*p*_^2^ = 0.75 and the corresponding RT significantly increased, *F*(2, 36) = 9.02, *p* < 0.001, η_*p*_^2^ = 0.33. Similar findings were observed for the correct rejections. As WM Load increased, the correction rejection rate significantly decreased, *F*(2, 36) = 6.05, *p* < 0.005, η_*p*_^2^ = 0.25 and RT significantly increased, *F*(2, 36) = 5.54, *p* < 0.008, η_*p*_^2^ = 0.24.

#### 3.1.2 VR WM

Performance was measured only in terms of accuracy. Response times were very long ranging from 10 to 15 s and consisted of a series of complex cognitive operations including preparation to respond, its initiation and its execution using both arm and hand movements. A one-way ANOVA with repeated measures on WM Load was run on the accuracy data. Accuracy significantly decreased as WM Load increased, *F*(3, 64) = 6.48, *p* < 0.001, η_*p*_^2^ = 0.23.

### 3.2 ERP data

#### 3.2.1 Standard ERPs

**FIGURE 2:**
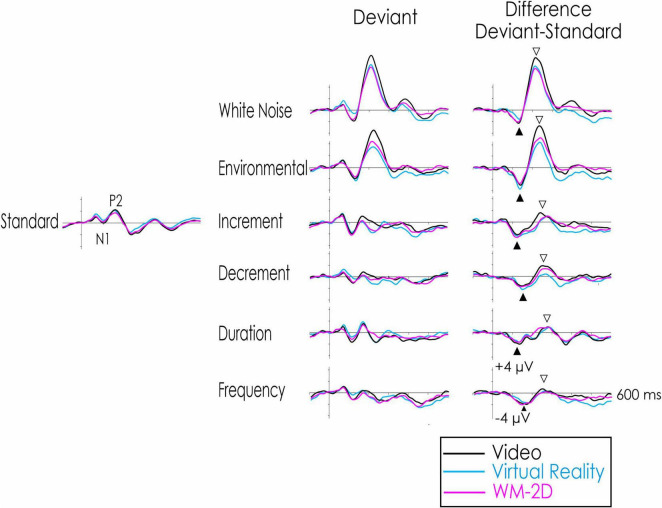
“Raw” standard and deviant ERPs (left and middle portions, respectively), and the deviant-standard difference wave (right portion). Negativity at the scalp relative to the nose reference is indicated by a downward deflection in this and all other Figures. The standard elicited only a small amplitude N1 at about 100 ms and a somewhat larger P2 at about 180 ms Neither the amplitude of N1 nor P2 significantly differed among the three task conditions. A DRN (filled upward arrow) is apparent for all deviants in the deviant-standard difference waveforms. The DRN was not significantly influenced by the different visual tasks. Only the white noise and environmental sound deviants elicited a significant P3a (downward open arrow). Its amplitude was significantly larger in the video condition. A significant P3a was not elicited by the other deviants in any of the tasks.

The DRN and P3a were quantified in a deviant–standard difference wave. The use of the difference wave assumes that experimental effects only affected the processing of the deviant. This assumption may not be true. Experimental effects might have affected the difference wave through either differential processing of either the deviant or the standard. The assumption that the processing of the standard was not affected by the visual task demands was therefore tested. The standard ERP waveforms are illustrated in the left-hand portion of Figure 2. The standard elicited a very small amplitude N1 because of the rapid rate of stimulus presentation. The amplitude of the N1 did not differ significantly among the three visual task conditions, *F* < 1 at both the frontal and central ROIs. Similarly, the amplitude of the following P2 was not significantly affected by the visual conditions, *F* < 1 at both ROIs.

#### 3.2.2 Deviant ERPs

##### 3.2.2.1 DRN

The deviant-standard difference waves are illustrated in the right portion of Figure 2. The six deviants elicited a DRN from 100 to 125 ms after stimulus presentation. The DRN was maximum over frontocentral areas and inverted in polarity at the mastoids. In the control condition, when participants were asked to watch a video and ignore the auditory stimuli, a significant DRN was elicited by all six deviant stimuli as determined by confidence interval testing. All six deviants were therefore included in the subsequent ANOVA procedure. Its amplitude varied significantly among the six deviants, *F*(2.8, 51.2) = 5.30, *p* < 0.01, η_*p*_^2^ = 0.23 at the frontal ROI. At the central ROI, the effect of the type of deviant failed to reach significance following Greenhouse-Geisser corrections, *F*(2.4, 44.0) = 2.47, *p* < 0.09, η_*p*_^2^ = 0.12 at the central ROI. Importantly, visual task demands did not however significantly affect the amplitude of the DRN, *F* < 1 at both the frontal and central ROIs. The Task × Deviant interaction was also not significant at either the frontal or central ROI, *F*(10, 180) = 1.23, *p* < 0.27, η_*p*_^2^ = 0.06 and *F*(10, 80) = 1.42, *p* < 0.17, η_*p*_^2^ = 0.07, respectively.

##### 3.2.2.2 P3a

**FIGURE 3:**
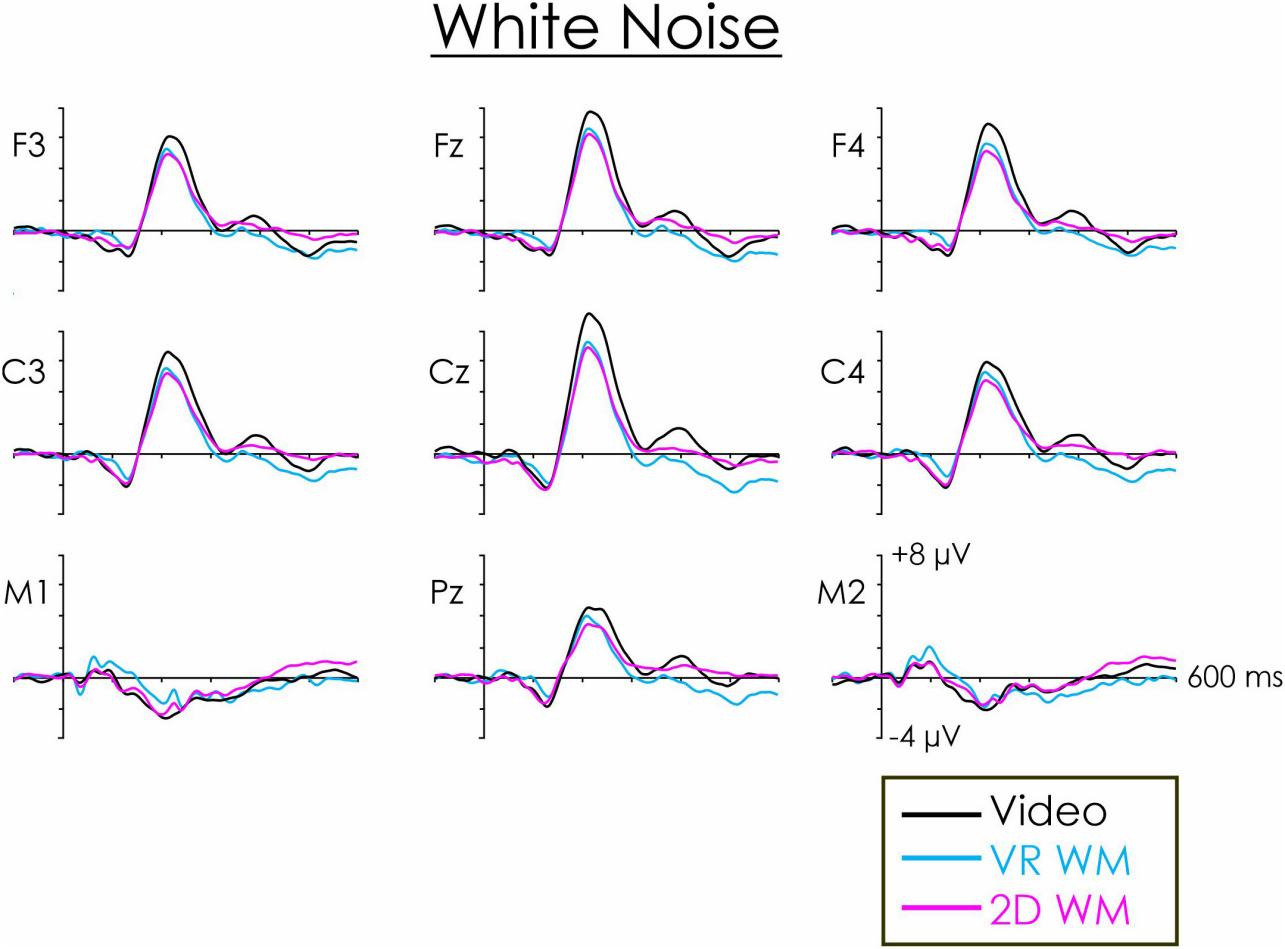
White noise deviant-standard difference wave across frontal, central, and parietal regions. A fronto-central maximum DRN is apparent at about 100–120 ms. It inverted in amplitude at the mastoids (M1, M2). This peak was followed by a P3a occurring at about 210 ms. At both central and frontal regions, the P3a was significantly larger when participants were asked to watch a video compared to when they were engaged in a VR WM or a 2D WM task.

**FIGURE 4:**
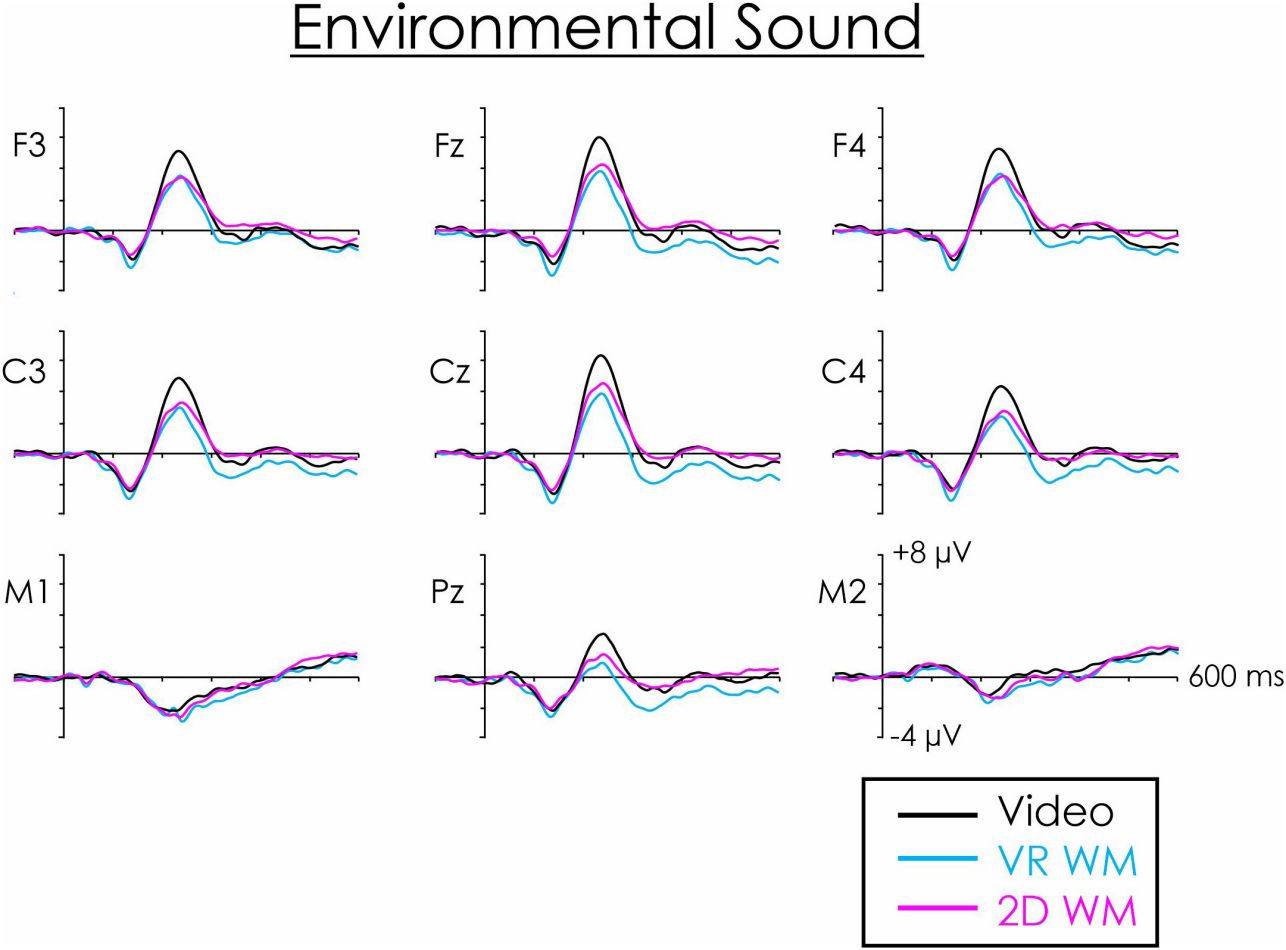
Novel environmental sound deviant-standard difference wave across frontal, central, and parietal regions. A fronto-central maximum DRN is apparent at about 120 ms. It inverted in amplitude at the mastoids (M1, M2). A P3a is also apparent at about 215 ms. At both central and frontal regions, the P3a was significantly larger when participants were asked to watch a video compared to when they were engaged in a VR WM or a 2D WM task.

A large positivity, the P3a, occurring between 200 and 225 ms was elicited by the white noise and novel environmental sounds in the control condition. The P3a was maximum over centro-frontal regions of the scalp. Confidence interval testing indicated that this positivity was only significant following presentation of the white noise (Figure 3) and environmental sound (Figure 4) deviants. The positivity was not significant for the remaining four deviants.

An initial ANOVA was run on all 6 deviants at the central ROI where it was largest. The main effect of deviant type was significant, *F*(5, 90) = 36.62, *p* < 0.001, η_*p*_^2^ = 0.68. Holm *post hoc* follow-up procedures indicated that the amplitude of the P3a for the white noise and environmental sound deviants was significantly larger (*p* < 0.01 in all comparisons) than for the remaining deviants. The amplitude of the P3a was very small and did not significantly differ (*p* > 0.05) among these 4 deviants.

**FIGURE 5:**
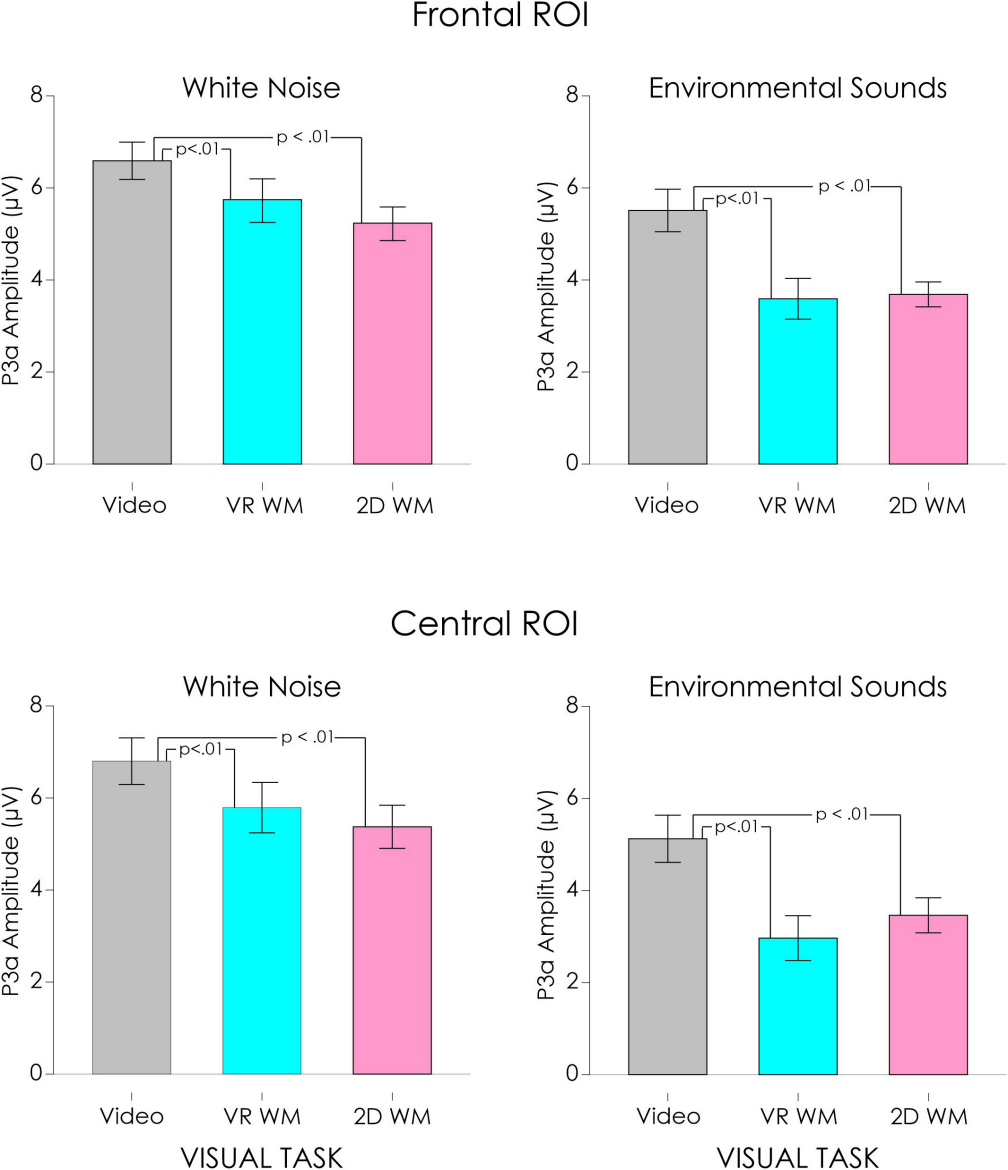
Mean P3a amplitude averaged across frontal and central ROI sites as a function of visual task for the white noise deviant (left) and the environmental sound deviant (right). Standard Error (SE) bars are also indicated error. P3a was significantly larger when participants were asked to watch a video compared to when they were engaged in a VR or a 2D working memory (WM) task.

A second ANOVA was therefore computed only for the white noise and environmental sound deviants. The mean values of the central ROI P3a as a function of visual task and type of deviant are illustrated in Figure 5. The main effect of visual task was significant, *F*(2, 36) = 15.75, *p* < 0.001, η_*p*_^2^ = 0.47. *Post hoc* testing revealed that the P3a was significantly larger when participants watched the video compared to carrying out either the VR WM task (*p* < 0.01) or the 2D WM task (*p* < 0.01). The effect tended to be larger for the environmental sound deviant (Figure 5), but the interaction between type of deviant and visual task was not significant, *F*(2, 36) = 2.81, *p* = 0.07, η_p_^2^ = 0.13. P3a differences between the VR WM and 2D WM tasks were not significant. Similar effects were apparent at the frontal ROI. The main effect of visual task was significant, *F*(2,36) = 11.70, *p* < 0.001, η_p_^2^ = 0.39. Again, *post hoc* testing revealed that the P3a was significantly larger when participants watched the video compared to carrying out either the VR WM task or the 2D WM task (*p* < 0.01 in both cases). The Deviant x Task interaction was again not significant, *F*(2, 36) = 2.63, *p* < 0.09, η_p_^2^ = 0.13.

##### 3.2.2.3 Scalp distribution maps

**FIGURE 6:**
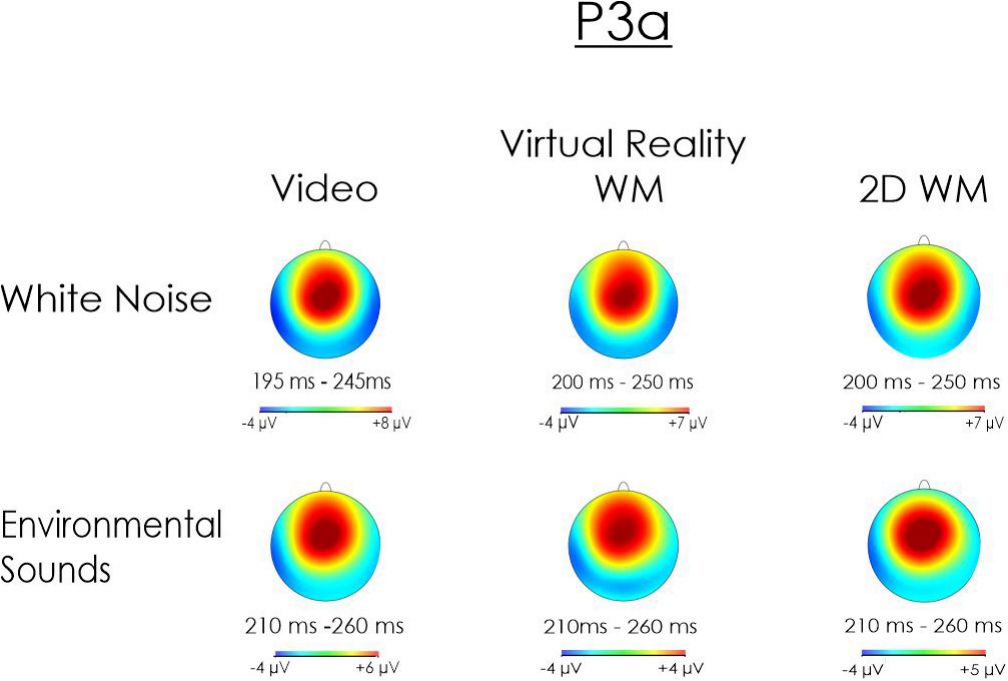
Spline maps of the P3a following presentation of the white noise and environmental sound deviants. The maps are displayed from a top of a flattened head that extends 20° below the Fp1-T7-Oz-T8-Fp2 circumference to show data from the most inferior electrodes (FT9, FT10, TP9, TP10). Note that the maps are scaled relative to the minimum and maximum amplitudes for each condition and type of deviant. The minima and maxima differ as a function of condition and type of deviant. The maps cannot therefore be used to determine where the condition and type of deviant influenced the absolute amplitude of the P3a. Rather the maps illustrate the difference in scalp topography. Thus, although the amplitude of the P3a was significantly reduced in the VR WM and 2D WM tasks, its scalp distribution was very similar to those observed when participants watched a video. The P3a was largest over centro-frontal regions of the scalp for the two deviants and in each of the Task conditions.

Exploratory spline scalp distribution maps (Perrin et al., 1989) of the P3a were also computed for the white noise and environmental sound deviants in the three visual task conditions (Figure 6). As can be observed, the P3a had a distinct centro-frontal positivity regardless of the type of deviant or the nature of the visual task. Thus, while the visual task did have a significant effect on the amplitude of the P3a, its scalp distribution did not appear to change across the three visual task conditions.

## 4 Discussion

When an individual’s attention is focussed on the processing demands of a cognitive task, certain rarely-presented but potentially highly relevant auditory stimuli that are not attended will be involuntarily processed. Such stimuli may cause a switch of attention from the relevant visual task-at-hand to the processing of the unattended auditory input. The salient features of the auditory input have prompted the central executive to shift processing priorities. The switching of attention may come at a cost. The switching of attention from the task-at-hand may result in a deterioration in performance on this task. In the present study, the extent of processing of the unattended auditory stimulus was quantified by recording event-related potentials (ERPs) following the presentation of these stimuli. Participants’ attention was directed to the demands of different visual tasks. Auditory stimuli were presented concurrently but these were irrelevant to the visual tasks and were to-be-ignored. The auditory sequence consisted of a frequently occurring standard stimuli and six different deviant stimuli. The deviants were created by varying different features of the standard. All deviants were expected to elicit a negativity occurring from 100 to 150 ms, the DRN, reflecting detection of stimulus change. The features of the white noise and environmental sound deviants represented a large extent of change from the standard stimulus and as such, were expected to elicit a later positivity occurring between 200 and 250 ms, the P3a. It is the P3a that is associated with attention capture and a switch of processing resources from the visual task-at-hand and toward the potentially more relevant processing of the auditory channel. Three different visual tasks were employed. The “gold standard” ERP procedure asks participants to watch a silent sub-titled video while ignoring the auditory sequence. This passive video was used as a control condition in the present study. A problem with the use of the video task is that the experimenter has little control over what the participant is actually “doing.” Participants might, for example, also eavesdrop on the auditory sequence. Two other visual task conditions were therefore also run. Participants were also presented with a WM task within a VR environment and in a third condition, a traditional WM task presented on a 2D monitor.

VR environments are claimed to be immersive, often evoking a distinct sense of presence in the virtual environment and a lack of awareness of events in the real, external environment (Sanchez-Vives and Slater, 2005). Such presence in a VR environment might thus prevent attention being switched from the relevant visual VR task to the irrelevant auditory channel. In the present study, an actual measure of presence was not taken. Nannipieri (2022) reports that as many as 40 different types of questionnaires have been administered purporting to measure the extent of perceived “presence.” Whether these questionnaires are able to measure an abstract concept such as presence has been seriously questioned. There are a number of issues. The questionnaires are typically administered after the session rather than during it and participants are asked to recall what they had experienced. Also, the questionnaires rely obviously on subjective report. Reviews by Marto and Gonçalves (2024) and Kukshinov et al. (2025) indicate that the many variants do not measure presence in the same way, and they ask different questions. Kusinov et al. note, “these numerous questionnaires often produce incomparable measurements of presence.” A limitation of the present study, is however, that it did not provide a measure of “presence” that is independent of the physiological measure, the P3a.

### 4.1 Performance data

Studies have reported that some participants experience different types of discomfort including motion sickness, nausea and headache within the VR environment. Such discomfort may have a pronounced adverse effect on performance. Such symptoms do vary with the testing procedures used within the VR environment. In a previous study using the same VR WM task, Kamal et al. (2025) administered the Simulator Sickness Questionnaire (SSQ) (Kennedy et al., 1993) to younger and older participants, none of whom were involved in the present study. Results indicated that only mild to moderate discomfort was experienced. For this reason, the SSQ was not administered in the present study. Participants were, however, asked if they had experienced discomfort within the VR environment. None reported adverse effects.

Performance on the VR WM and 2D WM tasks replicated many previous studies on the effects of WM load (Cowan, 2001; Luck and Vogel, 2013; Oberauer and Hein, 2012). As the number of objects needed to be maintained in memory (i.e., WM load) increased, accuracy of detection decreased in both versions of the WM task. Thus, in spite of the fact that both the stimulus and response parameters differed markedly between the two WM tasks, the effect of WM load was very similar. Importantly, the behavioral results can be used as evidence that the participant was indeed attentive to and engaged in the visual task-at-hand. On the other hand, the performance data cannot be used as evidence that the participants did not also attend the irrelevant auditory sequence. The ERP data were used to quantify the extent of processing of the unattended auditory stimuli.

### 4.2 ERP data

#### 4.2.1 DRN

As expected, the DRN was elicited by all deviants, reflecting the automatic detection of acoustic change. This DRN was also observed by Tavakoli and Campbell (2016) and Morrison et al. (2020) using a very similar multi-feature paradigm. The amplitude of the DRN was affected by the type of deviant that was presented. The purpose of this study was not to determine why deviant features can affect the amplitude of the DRN. Several studies have now demonstrated that the amplitude of the DRN/MMN varies directly with the extent of stimulus change in both oddball and multi-deviant paradigms (Sams et al., 1985; Tiitinen et al., 1994; Pakarinen et al., 2007; Duda-Milloy et al., 2019; Honbolygó et al., 2024). Moreover, Pakarinen et al. (2009) and Duda-Milloy et al. (2019) also used multi-deviant paradigms and observed that the amplitude of the DRN/MMN elicited by different deviants was directly related to behavioral detection rates for these deviants. On the other hand, Tavakoli and Campbell (2016) using the same deviants as those used in the present study did not observe a relationship between detectability of the deviants and the amplitude of the DRN.

There is some evidence that subtle reductions in the amplitude of the DRN/MMN can occur during exceedingly demanding visual tasks, but these effects are also dependent on the type of deviant (Näätänen et al., 1993; Muller-Gass et al., 2006). For this reason, several different types of deviants were used in the present study. It is also possible that the various visual tasks used in previous studies were not so demanding to prevent the participant from also sampling the auditory sequence. A VR WM task that was presumed to be highly demanding of cognitive resources was therefore used in the present study. The amplitude of the DRN elicited by the various deviants in the usual watching a video condition did not significantly differ from those elicited when the participant was engaged in a VR WM or a 2D WM task. This finding thus replicates those of many other studies examining the effects of visual task demands. Furthermore, this finding largely replicates other VR studies. Terkildsen and Makransky (2019) noted that a DRN (that they labeled as the “MMN”) elicited by a large frequency deviant (1,200 Hz standard, 2,000 Hz deviant) was reduced in those who experienced a large sense of presence within a VR environment compared to those who did not. In the present study, the frequency difference between the standard and deviant was much smaller (1,000 vs. 1,100 Hz). Grassini et al. (2021) did however also use a 1,200 and 2,000 Hz standard-deviant difference and also failed to observe the influence of VR presence on the elicited DRN. They did note that the Terkildsen and Makransky VR employed a horror-based VR game that may have induced a much stronger emotional reaction, fear, than their roller coaster ride. It is thus possible that modification of the DRN is only possible when a highly demanding cognitive task also activates strong emotional reactions to consume so much of the limited capacity system that very little is available for the processing of unattended auditory stimulus input.

#### 4.2.2 P3a

As expected, when participants watched the video, only certain deviant stimuli elicited a P3a. Tavakoli and Campbell (2016) and Morrison et al. (2020) also reported that only the white noise and environmental sound deviants elicited a P3a when a multi-feature paradigm was used. So powerful are the effects of these deviants that they may even elicit a P3a during an unconscious state, natural sleep (Tavakoli et al., 2019), perhaps alerting the sleeper to the occurrence of potentially highly relevant auditory input. Preventing such attention capture by the occurrence of such highly novel stimuli is therefore an onerous task. Remarkably, in the present study, the ability of these auditory deviants to capture attention and to distract attention away from the task-at-hand was reduced when participants were engaged in a WM task carried out in both the 2D and VR environments. Thus, while the DRN appears to reflect a largely automatic process, detection of acoustic change, this may not be the case for the P3a. The extent to which the occurrence of an unattended but highly novel auditory stimulus will interrupt the central executive’s ability to focus attention on a current task-at-hand thus appears to be dependent on the processing demands of this task. The switching of attention to the distracting event is therefore not a fully automatic, bottom-up process. It may also be at least somewhat influenced by top-down processes.

#### 4.2.3 Comparing VR and 2D WM tasks

The purpose of this study was to determine if attention capture by an irrelevant auditory stimulus can be reduced by the demands of a visual VR task. The amplitude of the P3a to very novel but unattended auditory stimuli was indeed reduced when participants were engaged in a WM task within the VR environment. Nevertheless, the reduction of the P3a was not unique to the VR condition. A reduction in P3a was also observed during another WM task carried out on a 2D monitor outside of the VR environment. It is therefore not clear if the reduction of the P3a within the VR environment was because of the unique demands of VR itself or a result of engagement in a WM task within this environment. The WM task parameters were quite different in the two conditions. The VR WM task was, of course, much more realistic involving a 3D high resolution head-mounted display. The stimulus and response parameters were also quite different. Thus, determining the extent to which the unique VR environment as opposed to the use of a WM task was responsible for the reduction of the P3a is very difficult to determine. In this context, Pappalettera et al. (2024) had participants actively attend to an auditory sequence to detect a rarely occurring target (deviant) stimulus. In different conditions, participants carried out the auditory task outside or within the VR environment. Within the VR environment, participants either were within an office or within an office but also interacting with avatars. The detection of a rare auditory stimulus elicited a P300. The P300 occurs later (around 300 ms) than the P3a and is associated with the active, rather than the passive detection of a rare target stimulus. The auditory P300 in the Pappalettera et al. (2024) was reduced in amplitude in the two VR conditions, even though the tasks did not require the use of WM. The P3a is usually recorded passively, when participants are not attending to the auditory sequence. Whether VR tasks not making large demands on WM will also modulate the passively-recorded P3a remains to be determined.

The nature of the WM tasks also requires further investigation. When participants are engaged in a *n*-back WM task presented on a 2D computer monitor, previous studies have shown the P3a may be modulated, but the results are inconsistent. In the *n*-back task, the participant is asked to determine whether the current stimulus matches the stimulus presented *n* trials earlier in the sequence. In the Muller-Gass and Schröger (2007) study, a 1-back memory task condition was run in addition to a perceptual task. Participants were asked whether the present short or long duration auditory stimulus was the same duration as the one that had preceded it. The pitch of the frequently occurring standard was at times changed to form a deviant, but the pitch change was irrelevant to the 1-back memory task. The distractor deviant resulted in poorer memory performance. These performance results were similar to those reported by Lavie (2005). In addition, a larger P3a was elicited when the participant had to decide whether the duration of the current auditory deviant was also presented in the previous trial (1-back condition) compared to when the participant had to decide about its duration (0-back condition). Thus, the *n*-back task seemed to *enhance* rather than protect against distraction. The effects of a distractor depend on several factors. In Muller-Gass et al. (2007) study, the auditory distractor occurred within an auditory *n*-back task. Other studies have used a visual *n*-back (Lv et al., 2010; SanMiguel et al., 2008). The deviant elicited a P3a that was reduced in amplitude when the *n*-back task was more demanding. On the other hand, Mahajan et al. (2020) did not find that *n*-back task difficulty had a significant effect on the amplitude of the P3a elicited by the auditory deviants. In the *n*-back studies, the presentation of an irrelevant auditory stimulus prior to the relevant visual stimuli is problematic. While the auditory stimuli were irrelevant to the visual *n*-back, they could still be used as a warning signal or as a cue to predict the subsequent occurrence of the visual target (Baragona et al., 2025; Parmentier, 2014). Thus, attending to the auditory sequence could improve performance on the visual WM task. As such, in some conditions, differences in the amplitude of the P3a may have been a result of passive compared to active processing of the deviant. Comparing the P3a elicited in different types of WM tasks (delayed match-to-sample versus n-back tasks) is therefore difficult. Compounding the issue is the fact that while processing demands in the two types of WM task may be very different, the placement of the actual auditory distractor is also quite disparate. In the current delayed match-to-sample tasks, the auditory stimuli occurred in the “background” and provided no information about the visual stimuli. The P3a was therefore elicited passively. In most *n*-back studies, the auditory stimuli are presented immediately prior to the occurrence of the visual stimuli to-be-remembered. They could thus be informative, providing a cue to the imminent occurrence of the task relevant visual stimuli. The P3a might therefore have been recorded actively.

### 4.3 Disentangling VR and 2D WM tasks

The purpose of the present study was to also determine if the amplitude of the P3a could be modulated by task demands. This goal was successful. Although both the VR and 2D tasks did involve the use of WM, there were wide methodological discrepancies in stimulus and response procedures. Our study was not designed to determine whether VR *per se* or the use of a WM task was responsible for the reduction in the P3a. Future studies could construct a VR WM task with parameters that are much more similar to those of a standard 2D task. However, this might also result in a large reduction of the immersiveness and sense of presence in the VR environment. To determine whether the reduction of the P3a within the VR environment was because of its unusual demands for cognitive resources or because the VR task involved processing associated with WM will require the inclusion of other control conditions. A highly demanding non-WM task could also be used within the VR task for this purpose. It would also be essential to include non-demanding VR conditions. For example, Lier et al. (2020) had participants watch a video within a VR environment. Painful electric shocks were occasionally delivered but these were irrelevant to the content of the video. The electric shock did elicit a large P3a in a control condition (watching a static image). Its amplitude was, however, much reduced when the participant watched the video.

In the present study, the reduction of the P3a in both WM tasks provided evidence of decreased involuntary attention capture, often associated with distraction. However, conclusions about actual distraction would have required an independent measure of performance on the assigned WM tasks. In many ERP studies, the deterioration in performance as measured by accuracy of responding or response times provides such an independent measure of distraction. A measure of distraction could have been included in the present study by running two other VR WM and 2D WM conditions in which the auditory stimuli were not presented. Presumably, performance on these tasks would have deteriorated when the auditory sequence was presented. These additional tasks would, of course, have increased the duration of an already long testing time. An independent performance measure of distraction by irrelevant auditory has been included in some *n*-back studies. The auditory stimuli can be synchronized to occur just prior to the presentation of the visual stimuli. Presumably if attention is switched to the auditory stimuli, a P3a should be elicited and performance on the visual *n*-back should deteriorate. However, as mentioned previously, such synchronization may result in a confound, the apparently irrelevant auditory stimuli could now act as a relevant warning signal rather than being a distractor. Moreover, even if these confounds could be overcome (see Wetzel et al., 2013), synching of the auditory and visual stimuli in a delayed match-to-sample paradigm would remain very problematic. Models of the MMN propose that the MMN reflects the output of a rapidly-fading sensory memory. The deviant must occur before the memory representation for the standard fades. The duration of sensory memory has been estimated to last from perhaps 2 to 10 s (Sabri and Campbell, 2001; Sams et al., 1993). In the VR WM task, a trial was initiated much more slowly than this.

The influence of task demands on the P3a could also be further explored by examining the influence of WM load. Performance was poorer as WM load increased. Trials could therefore be sorted according to WM load. When the load was high (e.g., 4 items to-be-remembered), it might be expected that the P3a would be reduced compared to when load was low (e.g., 1 item to-be-remembered). Unfortunately, the analysis of the influence of load would probably not permit the use of a multi-feature paradigm. In the present study, a total of 154 of each type of deviant was presented in each condition. Had there been sorted by the four WM loads, then only about 40 trials would have been available for averaging. This low number would have been insufficient to permit reduction of the background EEG noise to permit the ERP signal to emerge.

## Data Availability

The datasets presented in this article are not readily available because we do not have ethical approval to share data. Requests to access the datasets should be directed to cassandramorrison@cunet.carleton.ca.
